# Horizons in Human Aging Neuroscience: From Normal Neural Aging to Mental (Fr)Agility

**DOI:** 10.3389/fnhum.2022.815759

**Published:** 2022-06-29

**Authors:** K. Richard Ridderinkhof, Harm J. Krugers

**Affiliations:** ^1^Department of Psychology, University of Amsterdam, Amsterdam, Netherlands; ^2^Amsterdam Center for Brain and Cognition (ABC), University of Amsterdam, Amsterdam, Netherlands; ^3^SILS-CNS, Faculty of Science, University of Amsterdam, Amsterdam, Netherlands

**Keywords:** aging, cognitive agility, mental fragility, neural decline, hippocampus, prefrontal cortex (PFC)

## Abstract

While aging is an important risk factor for neurodegenerative disorders such as Alzheimer’s disease and Parkinson’s disease, age-related cognitive decline can also manifest without apparent neurodegenerative changes. In this review, we discuss molecular, cellular, and network changes that occur during normal aging in the absence of neurodegenerative disease. Emerging findings reveal that these changes include metabolic alterations, oxidative stress, DNA damage, inflammation, calcium dyshomeostasis, and several other hallmarks of age-related neural changes that do not act on their own, but are often interconnected and together may underlie age-related alterations in brain plasticity and cognitive function. Importantly, age-related cognitive decline may not be reduced to a single neurobiological cause, but should instead be considered in terms of a densely connected system that underlies age-related cognitive alterations. We speculate that a decline in one hallmark of neural aging may trigger a decline in other, otherwise thus far stable subsystems, thereby triggering a cascade that may at some point also incur a decline of cognitive functions and mental well-being. Beyond studying the effects of these factors in isolation, considerable insight may be gained by studying the larger picture that entails a representative collection of such factors and their interactions, ranging from molecules to neural networks. Finally, we discuss some potential interventions that may help to prevent these alterations, thereby reducing cognitive decline and mental fragility, and enhancing mental well-being, and healthy aging.

## Introduction

Beyond the age of roughly 55, progressing age is a risk factor for the development of neurodegenerative disorders, among which Alzheimer’s disease, Parkinson’s disease, and stroke are the most common (Krishnamurthi et al., 2013; Kalia and Lang, 2015; Scheltens et al., 2016; Aarsland et al., 2017). However, advancing age is also the main risk factor for mental fragility (the susceptibility to loss of mental agility and vigor) and the decline of mental abilities during “normal” or “successful” aging (in individuals without clinical diagnoses of, for example, Alzheimer’s disease or Parkinson’s disease).

Aging is often characterized by a progressive loss of neurophysiological function, leading to impaired cognitive capacities. Yet, older adults may in fact age “successfully” despite physical or cognitive limitations, as long as these have little influence on their well-being and quality of life (Young et al., 2009). The World Health Organization defines successful aging as the process of developing and maintaining the functional ability and competence that enables mental well-being in later life^1^. Here we review various hallmarks of neural aging vis-à-vis the “normal” deterioration of mental ability and agility. The literature from the *neurobiological point of view* on normal aging tends to focus on cellular and molecular mechanisms, processes, antecedents, and cognitive consequences of neural aging. In comparison with this focus on the micro-level, the literature from the *neuropsychological point of view* on normal aging tends to focus more on the macro-level including relationships between specific cognitive functions, and functional and structural integrity (activation and connectivity) within and between large-scale neural networks.

The aim of this review is four-fold. First, we aim to bridge the gap between these two largely segregated sets of literature, and provide a selective review and synthesis of patterns of normal aging across levels: from molecular to cellular and network changes to behavioral/mental abilities and agility. We purposefully focus on *normal* rather than pathological aging (such as seen in neurodegenerative diseases). Second, we aim to show that *intra*-neuronal and *inter*-neuronal mechanisms and antecedents of normal neural aging form a complex of intricately interconnected factors that engage in complex interactions that are so widespread that, beyond studying the effects of these factors in isolation, considerable insight may be gained by studying the larger picture that entails a representative collection of such factors in interaction, ranging from molecular to neuronal networks. We argue that a full mechanistic understanding of mental aging cannot be reached by focusing on a single neurobiological factor, but should instead be studied by focusing on reciprocally causally connected factors. Third, we aim to illustrate how the interplay of these generic neural factors connects to cognitive decline, with a focus on the hippocampus and prefrontal cortex. Normal neural aging in the complex of factors that tap into these brain areas results in declines in memory function, stress regulation, and resilience. Fourth, we discuss the effects of interventions at molecular, cellular, network, and cognitive levels: factors that influence metabolism (calorie restriction, exercise) as well as nonmetabolic factors (microglia replacement, microbiota, certain chemicals, sleep hygiene, and chronic stress). The discussion of such interventions will underscore that their effects extend to the entire complex of neural factors (even if they appear to target specific factors in isolation), and will help to understand how such effects extend from molecular to cognitive levels.

We propose that a richer understanding of these relationships will help push the field forward, which is now more urgent than ever since the mental and physical well-being and health status of vulnerable seniors present massive and urgent concerns not only to aging individuals and their caretakers but also to our society at large. Indeed, active and healthy aging is currently a top priority of the World Health Organization, which in fact has dubbed the present age as the decade of healthy aging. This is emphasized by the notion that the proportion of individuals aged 65+ in Western societies is rising to >25%, with a concomitant decline in mental health and well-being.

We first discuss briefly *why* humans age (from an evolutionary perspective) in order to better understand *how* human brains age (from a biological perspective). After discussing the hallmarks of cellular and molecular deterioration, we evaluate how these neural changes alter the (functional) integrity of larger-scale networks. Using the hippocampal system and prefrontal cortex as representative examples, we review how such changes ultimately affect cognitive efficiency (from a psychological perspective). Next, we discuss the effects of interventions that target molecular and cellular factors of normal neural aging, and highlight their wide-ranging effects on cognitive functions. Finally, we summarize and prepare some outstanding questions that may help further to understand the underpinnings of mental wellbeing during aging, and possible intervention strategies.

To preview, we discuss that normal aging is accompanied by various intra-neuronal molecular and cellular alterations, such as metabolic alterations, calcium homeostasis, DNA damage, and oxidative stress that disturb cellular function which in turn may alter and impact cognitive function. Importantly, these alterations are often interrelated and in synergy may enhance cellular and inter-neuronal network perturbations in relation to cognitive decline. Interventions that target metabolic (calorie restriction, exercise) and non-metabolic processes (stress, sleep, microbiome) may be promising avenues for enhancing mental well-being and agility, thereby reducing mental fragility.

## Why Human Brains Age: An Evolutionary Perspective

Animals are not programmed to die. Rather, they are programmed for survival in the face of a variety of challenges to their survival or integrity at the cellular and molecular levels. An extensive repertoire of cellular and molecular protection mechanisms prioritizes short-term over long-term maintenance and repair systems. When facing many threats to survival, limited energy supplies are geared toward procreation rather than towards continued bodily maintenance. This evolutionary principle may help to understand why people age and die. As people live their lives, some of the many challenges to the integrity of their bodily tissues cannot be met adequately, and the aging process is associated with the gradual accumulation of unrepaired deficiencies, physiological impairments, and degenerative functional consequences.

Germ cells generate oocytes and sperm cells, and thereby transmit genes to the next generation. Liberated from the need to support reproduction, somatic cells have evolved into specialized cells, such as nerve cells, which are equipped for the conduction of chemical and electrical signals. This specialization yields extraordinary advances in terms of differentiation, but also came at the expense of aging and degeneration. Organisms that lack specialized somatic cells, such as the hydra and other mollusks, don’t age, and die only through extreme environmental changes such as predation, starvation, or extreme temperature changes. Organisms that have evolved specialized somatic cells however do age and die. These somatic cells are disposable (Kirkwood, 2001): when their repair and maintenance become too costly, energy is diverted to procreation. The evolution of specialized soma cells came with aging.

DNA carries the genetic information of cells and is essential for the generation of proteins and cellular function. Damage to DNA may occur, but mechanisms have been developed to reduce DNA damage. Occasionally, however, DNA damage fails to get repaired correctly which can lead to lasting alterations in cellular function (Maynard et al., 2015). This persisting damage causes corruption of the information coded in our DNA, resulting in for example random variations in how genetic instructions translate into proteins and protein transport, in the signals that are sent to other cells, in the read-out of signals from other cells. Once these changes become too detrimental to cell-survival the cell may die (Maynard et al., 2015).

Via the process of evolutionary pressure, DNA damage does not interfere with vital functions too much until past our reproductive years. Evolution takes little interest in later life. Still, cells from long-lived animals are generally better at DNA repair than cells from short-lived animals. As animals adapted to their environments and were successful in surviving the dangers they encountered, it became worthwhile to invest in mechanisms that help to promote longevity, such as better DNA repair mechanisms. These adaptations made the soma less disposable and, consequently, life span increased. Some species (including humans, orcas, elephants, and giraffes) even developed a prolonged post-reproductive state of life that proved evolutionary adaptive (such as female menopause; Muller and Harris, 2021). Such older females can help to promote the survival chances of their offspring’s offspring by providing protection and other beneficial conditions (such as knowledge about food and water resources in times of scarcity).

While animals age, their brains are also subject to the aging process. Brains have evolved as organs specialized in sustaining the organism’s vital bodily functions and needs. The brain supports the organism by perceiving the state of the external world as well as its own internal milieu, and by acting to meet its needs vis-à-vis these perceived states. Thus, the brain organizes and coordinates action geared towards goals to obtain and sustain agreeable states (health, food, drink, sex, happiness, connectedness, affirmation) and to avoid aversive states (pain, anxiety, frustration, social isolation, depreciation) (Ridderinkhof, 2014). Throughout evolution, mental functions have become more acutely instrumental to these aims; memory (storing relevant perceived information), decision-making, perception-action selection, planning, and the ability to navigate exploitation/exploration, consistency/flexibility, and accuracy/speed balances. In the brain, just as in other body parts, challenges to cellular or molecular integrity result in errors and damage, with mental dysfunction as an ultimate consequence.

In this article, we focus on understanding normal neural aging and its relation to age-related changes in mental agility and fragility. The appreciation of the principle that organisms are programmed to survive rather than die, may help to understand and appreciate that the aging process is malleable. Aging is accompanied by the accumulation of subtle damage and functional impairments in body cells. It may, therefore, be potentially possible to influence the aging process by choices that impact exposure to damage, or our ability to cope with it, as will be discussed later. In the next sections, we discuss several hallmarks of cellular and molecular deterioration and subsequently evaluate how this induces neural decline and the integrity and function of larger-scale networks.

## How Human Brains Age: Intra-Neural and Inter-Neural Hallmarks of Neuronal Deterioration

### Molecular and Cellular Hallmarks of Brain Aging

The time-dependent accumulation of neural damage is widely considered to be a generic causal factor underlying neurophysiological aspects of mental aging (Kirkwood, 2005; Vijg and Campisi, 2008; Cagan et al., 2022). However, this accumulation of damage is but one factor in a multifaceted system of hallmarks of intra-neural aging and their interconnections that together determine the “aging phenotype”, and that together constrain the possibilities to intervene exogenously to delay aging. The hallmarks of brain aging at the *cellular* and *molecular* levels (López-Otín et al., 2013; Mattson and Arumugam, 2018) form a complex picture. As will be discussed in more detail below, these hallmarks include multiple factors that can interact with each other (Mattson and Arumugam, 2018). Here we provide an overview of these hallmarks, followed by more detailed and fully referenced subsections for the more specialized reader. These subsections highlight various hallmarks of intra-neural aging, and several interconnections between these hallmarks, that together form a multifaceted system that determines the aging phenotype. This pattern can be summarized as follows, the dimensions of metabolism on the one hand, and damage and waste on the other.

Among the complex set of factors that are related to normal neural aging, reduced mitochondrial fitness is densely interconnected with many other such factors: it affects glucose metabolism, cellular Ca^2+^ homeostasis, protein deacetylases of sirtuins, accumulated oxidative stress, reactive oxygen species (ROS), and damaged DNA. Glucose metabolism in nerve cells can be impaired during aging as an indirect result of oxidative stress. The intracellular signaling pathway of insulin and Insulin-like growth Factor (IGF)-1 informs cells of the presence of glucose; with normal aging, downregulation of the insulin and IGF-1 signaling pathways serves to reduce the rate of cell growth and metabolism, and hence increase longevity. Insulin resistance is associated with poorer cognitive function during aging. Aging increases the Ca^2+^-dependent after hyperpolarization, Ca^2+^ currents and spikes, and release from intracellular stores. Persistent increase in intracellular Ca^2+^ levels impacts activation of proteases, lipases, tau hyperphosphorylation, microtubule depolymerization, and synaptic function, which can contribute to cognitive decline.

During the course of normal neural aging of cellular function, the integrity and stability of DNA in the mitochondria and nucleus are continuously challenged by endogenous threats, including DNA replication errors and ROS. In healthy nerve cells, oxidative DNA damage can be repaired, but while the amount of damaged nuclear and mitochondrial DNA increase during aging, DNA repair pathways deteriorate. Telomeres are particularly susceptible to age-related accumulation of DNA damage and their replication through telomerase is increasingly imperfect with aging.

Aging is also characterized by the neuronal accumulation of dysfunctional and aggregated proteins and mitochondria. This accumulation results in part from an oxidative imbalance: upregulated production of ROS and downregulated antioxidant defenses. Oxidative stress can impair the autophagic function of proteasomes and lysosomes, and is linked to cognitive decline. Aging is accelerated when resistance against ROS and the ability to remove oxidatively damaged molecules are compromised. On the other hand, ROS can trigger proliferation and survival in response to stress by activating compensatory homeostatic responses. Removing damaged and dysfunctional molecules, defective organelles, and misfolded proteins is important for neurons to maintain their functional integration into neuronal networks. Damaged cellular constituents are transported to lysosomes or proteasomes for further degradation. Autophagic and proteasomal waste management in nerve cells is impaired during aging. Apoptosis may be triggered by telomere shortening and other sources of DNA damage.

Inflammageing is a low-level chronic inflammation characterized by an increase of pro-inflammatory cytokines as aging progresses. Astrocyte senescence results in the elevated expression of pro-inflammatory cytokines. Inflammation activates neural microglia, and activated microglia can clear cellular debris. As the organism ages, the microglia have smaller areas they can patrol to clear debris, and show a significantly slower reaction to acute brain injury. Moreover, in the aged brain, microglia often exhibit an activated pro-inflammatory state which can enhance oxidative damage. Healthy regulation of the activation of immune cells contributes to neuronal stress resistance.

Taken together, there is ample evidence that in the course of aging, the brain is subjected to various metabolic and oxidative challenges that render neurons vulnerable to impaired function. At the same time, the potential capacity of appropriate defense mechanisms is reduced. This may over time result in cognitive and functional alterations, that can vary between individuals, e.g., depending on “environmental” experienced challenges and defense mechanisms, which may more be genomic by nature.

In Section “Compromised Neuronal Activity and Network Integrity”, we discuss in more detail how the interplay of these neural factors connects to and may alter cognitive decline, with a focus the hippocampal formation. Normal neural aging in the complex of factors that tap into the hippocampal system results in impairments, in memory function, stress regulation, and resilience. Assessing the relationships between compromised neuronal activity and network integrity can help close the gap between neurobiological aging at the molecular and cellular levels on the one hand, and the decline of complex cognitive functions on the other. For the interested reader, however, we first provide more detailed subsections on the cellular and molecular hallmarks of neural aging.

#### Mitochondrial Dysfunction

Mitochondria are organelles whose function is essentially to produce adenosine triphosphate (ATP) molecules that provide energy to support electrochemical neurotransmission, cell maintenance, and cell repair (Mattson, 2009). Aging is associated with oxidative damage to mitochondrial DNA, altered mitochondrial shape, and reduced mitochondrial function (Santos et al., 2013; Stahon et al., 2016; Sun et al., 2016; Morozov et al., 2016; Paradies et al., 2017). Mitochondrial fitness is inter-related to many other factors that are related to normal neural aging (Sebastián et al., 2017). It affects cellular metabolism (Tavallaie et al., 2020), cellular Ca^2+^ homeostasis (Raefsky and Mattson, 2017), protein function such as deacetylases of the sirtuin family (Fang et al., 2017), accumulated oxidative stress (Harman, 1956), and DNA damage (Bratic and Larsson, 2013). These changes may eventually lead to alterations in brain function (Mattson and Arumugam, 2018), but also peripheral metabolic complications such as hypertension or obesity that can affect mental wellbeing (Vaughan and Mattison, 2016; Lahera et al., 2017).

#### Dysregulated Glucose Metabolism

Aging has been associated with elevated levels of both glucose and insulin after oral glucose challenge testing (glucose intolerance, DeFronzo, 1981; Kalyani and Egan, 2013). Glucose metabolism in nerve cells has been reported to be impaired during aging as a result of a compromised ability to increase glucose transport in response to insulin (Goyal and Dawood, 2017; Mattson and Arumugam, 2018). In addition to impaired glucose transport, insulin resistance has been associated with poorer cognitive function during aging (Thambisetty et al., 2013) but also constitutes a major risk factor for diabetes, cardiovascular disease, and stroke, although older adults with diabetes and altered glucose status may represent a vulnerable subset of the population at high-risk for complications and adverse geriatric syndromes including physical problems, functional disability, frailty, and early mortality (Kalyani and Egan, 2013).

#### Aging and Sirtuins

Sirtuins are a class of proteins that regulate gene activity in cells. The function of Sirt1 (the most extensively studied member of the sirtuins family) is to deacetylase various proteins and substrates, thereby modifying the physical structure of certain regions of the genome, thus modifying gene expression (Chang and Guarente, 2014). As such, Sirt1 is involved in many physiological functions, among which metabolism. Oxidate stress and reactive oxygen species (ROS, see Section “Accumulation of Oxidatively Damaged Molecules”) have negative effects on the activity of Sirt1 (Braidy et al., 2011), thereby impacting the protection of neurons when exposed to neurotoxic damage since Sirt1 enhances the activity of antioxidant enzymes and reduces the production ROS (Chan et al., 2017). This is relevant for aging since Sirt1 activity decline with aging (Rahman and Islam, 2011). Interestingly, transgenic overexpression of mammalian SIRT1 improves aspects of health during aging (but does not increase longevity; Herranz et al., 2010).

#### Accumulation of Oxidatively Damaged Molecules

Aging is characterized by the neuronal accumulation of dysfunctional and aggregated proteins and mitochondria (Lin et al., 2007; Ghosh et al., 2011). This accumulation results in part from an oxidative imbalance: upregulated production of ROS and downregulated antioxidant defense mechanisms (Maynard et al., 2015). Reactive oxygen species are a natural byproduct of cellular processes; in nerve cells, most ROS are the superoxide anion radical and nitric oxide, generated in response to elevated intracellular Ca^2+^ levels (Halliwell, 2007). When ROS cannot be buffered, a misbalance occurs in which they become toxic and cause damage to the cell and its DNA. Oxidative stress can impair the autophagic function of proteasomes and lysosomes (Butler and Bahr, 2006; Zhang et al., 2017), and is linked to cognitive decline (Kandlur et al., 2020). Aging is accelerated when resistance against ROS and the ability to remove oxidatively damaged molecules are compromised (Melov et al., 1999; Paul et al., 2007). On the other hand, ROS is important for neuronal development (Oswald et al., 2018) and can trigger proliferation and survival in response to stress (Sena and Chandel, 2012). Thus, ROS may act as a stress-elicited survival signal that activates compensatory homeostatic responses (López-Otín et al., 2013; Oswald et al., 2018; Stefanatos and Sanz, 2018). With advancing age, however, levels of ROS increase as cellular stress and damage increase and can aggravate rather than alleviate damage (Stranahan and Mattson, 2012). Thus, a balance in ROS is important for proper cellular function.

#### Inflammation

The nervous system has co-evolved with the immune system, and inflammation is common in brain aging. Inflammageing is a term coined by Franceschi et al. (2006) to describe a state or a process seen in humans as they age. It is described as a low-level chronic inflammation that is present in the organism, characterized by an increase of pro-inflammatory cytokines as aging progresses. It is considered a form of *sterile* inflammation because it is not a reaction to any pathogen or injury (Chinta et al., 2015), and its symptoms are subclinical (Fülöp et al., 2019).

Microglia and astrocytes are activated in the brain in relation to inflammation, and activated microglia can clear cellular debris. Microglia communicate with other cells by releasing signaling molecules called cytokines (Hefendehl et al., 2014). Important pro-inflammatory cytokines are Tumour Necrosis Factor alpha (TNF-α), Interleukin-1 beta (IL-1β), and Interleukin-6 (IL-6). While persistent activation of microglia may be harmful, microglia have also been implicated in synaptic plasticity and learning and memory (Di Benedetto et al., 2017; Sanguino-Gómez et al., 2021).

Astrocytes become activated by and react to cytokines produced by microglia, subsequently releasing cytokines of their own which can amplify the microglial immune reaction (Calabrese et al., 2018). Age-related changes in astrocytes have been linked to elevated expression of pro-inflammatory cytokines (Chinta et al., 2015; Calabrese et al., 2018; Rea et al., 2018; Fülöp et al., 2019) and in deterioration of the Blood-Brain Barrier (BBB; Chinta et al., 2015), which can increase inflammation by allowing pro-inflammatory molecules or cells from the periphery to pass into the central nervous system.

In their normal resting state, microglia patrol the environment and participate in clearing debris and neuronal support functions (Di Benedetto et al., 2017). As the organism ages, the reaction of microglia toward acute brain injury is altered (Hefendehl et al., 2014). Moreover, in the aged brain, triggered by cytokines like TNF-α and IL-1β, microglia often exhibit an activated pro-inflammatory state (Di Benedetto et al., 2017). Prolonged activation of microglia may relate to (chronic) inflammageing. Thus, while healthy regulation of the activation of immune cells contributes to neuroplasticity and neuronal stress resistance, aberrant activation may result in synaptic alterations and age-related functional impairment (Da Mesquita et al., 2021; Sanguino-Gómez et al., 2021).

#### Deficiencies in Insulin and Insulin-Like Growth Factor

The intracellular signaling pathway of insulin and insulin-like growth factor 1 (IGF-1) informs cells of the presence of glucose. Genetic polymorphisms or mutations that reduce the functions of IGF-1 or insulin receptors have been linked to longevity in humans (Fontana et al., 2010; Barzilai et al., 2012). IGF-1 levels decline during normal aging (Schumacher et al., 2008). Interestingly, mice that show overexpression of the tumor suppressor *PTE* exhibit a down-regulation of the insulin and IGF-1 signaling pathways, improved mitochondrial oxidative metabolism, and increased longevity (Ortega-Molina et al., 2012). Downregulation of the insulin and IGF-1 signaling pathways serves to reduce the rate of cell growth and metabolism, and hence increase longevity, in the context of normal aging as well as systemic damage (Garinis et al., 2008).

#### Dysregulation of Neuronal Calcium Homeostasis

Calcium (Ca^2+^) is of fundamental importance for neuronal function given its role among other ynaptic transmission and synaptic plasticity (Brini et al., 2014). Ca^2+^enters the cell *via* membrane receptors (such as NMDA receptors) and voltage-dependent calcium channels. In addition, Ca^2+^ can be released from intracellular stores, such as the endoplasmic reticulum (ER) and mitochondria. Intracellular/cytosolic Ca^2+^ levels are tightly regulated *via* Ca^2+^-binding proteins and for example by its uptake and release by mitochondria, ER, and *via* the Na^+^/Ca^2+^ exchanger in the plasma membrane (Brini et al., 2014).

Various lines of evidence suggest that sustained disruptions of calcium homeostasis are involved in brain aging. For example, aging increases the Ca^2+^-dependent after hyperpolarization, Ca^2+^spikes, Ca^2+^ currents, and release from intracellular stores (Thibault and Landfield, 1996; Foster and Kumar, 2002; Toescu et al., 2004; Kumar et al., 2009; Alzheimer’s Association Calcium Hypothesis Workgroup, 2017). Persistent increase in intracellular Ca^2+^ levels may impact a.o. activation of proteases, lipases, tau hyperphosphorylation, microtubule depolymerization; and synaptic function, which can contribute to cognitive decline (Kumar et al., 2009).

#### DNA Damage and Impaired DNA Repair

The accumulation of DNA damage throughout life, and especially in later life, is among the most common hallmarks of aging (Moskalev et al., 2013). During the course of normal aging , the integrity and stability of DNA in the mitochondria and nucleus are continuously challenged by endogenous threats, including DNA replication errors and exposure to ROS (Hoeijmakers, 2009). In order to minimize these alterations, organisms have evolved a complex network of DNA repair mechanisms that are able to cope with most of the damages inflicted to nuclear DNA (Lord and Ashworth, 2012). That is, in healthy nerve cells, damaged DNA bases are rapidly removed and replaced by the coordinated activities of proteins in DNA repair pathways, which is critical for the repair of oxidative DNA damage (Mattson and Arumugam, 2018). However, the amount of damaged nuclear and mitochondrial DNA increases during aging, whereas DNA repair mechanisms deteriorate. This accumulation may be particularly prevalent in the brain given the low DNA repair capacity in post mitotic brain tissue and contribute to aging (Maynard et al., 2015). Indeed, accumulation of damage to mitochondria and mitochondrial DNA may reduce physiological dysfunction and eventually lead to pathology (Maynard et al., 2015).

#### Reduced Telomere Length

Telomeres are the regions at the ends of the linear chromosomes and are composed of DNA and proteins (Hou et al., 2020). Telomeres become shorter as cells divide unless mechanisms are present to prevent attrition. While telomeres diminish with each cell division, the enzyme telomerase is able to repair this thereby preventing the telomeres from declining (Liu et al., 2018). Telomere replication is imperfect however and after multiple cell divisions, the DNA becomes compromised, potentially leading to age-related diseases. Shortening of telomeres has been implicated in aging, and telomeres are particularly susceptible to age-related accumulation of DNA damage (Blackburn et al., 2006; De Jesus et al., 2012; Hewitt et al., 2012; Turner et al., 2019). Interestingly, telomerase reactivation (and telomere elongation) may reverse age-related pathology in mice (Jaskelioff et al., 2011; Maynard et al., 2015).

#### Impaired Molecular Waste Disposal

In order to maintain function, all body parts need to continually remove metabolic waste to preserve their homeostasis throughout the lifespan of an organism. Removing damaged and dysfunctional molecules, defective organelles, and misfolded proteins is particularly important for neurons to maintain their functional integration into neuronal networks. Damaged cellular constituents are transported to lysosomes or proteasomes for further degradation. For instance, microglia engulf misfolded proteins and clear α-synuclein *via* autophagy (Choi et al., 2020). The autophagic and proteasomal waste management in nerve cells is impaired during aging (Graham and Liu, 2017; Kerr et al., 2017). With increasing age, fewer autophagosomes are produced that more often have distorted shapes (Stavoe et al., 2019). Moreover, neural proteasome activity decreases with age (Keller et al., 2002; Koga et al., 2011), and, together with age-related neural lysosome dysfunction, may result in the accumulation of cellular debris (Zhang et al., 2017) resulting in deficient neuronal function.

### Compromised Neuronal Activity and Network Integrity

As will be discussed in more detail below, neural aging at the structural and inter-neuronal level pertains to: (1) impairments in neurogenesis, including nerve cell senescence and stem cell exhaustion; (2) altered neuronal integrity, activity, plasticity, and communication; and (3) aberrant neuronal network activity and connectivity. Once more, we first provide an overview of these age changes, followed by more detailed and fully referenced subsections for the more specialized reader.

During adulthood, the process of neurogenesis has been implicated in learning and memory. Consequently, apoptosis and diminished neurogenesis in later life resulting from neural stem cell exhaustion and inefficient clearance of senescent cells may result in cognitive decline. At the more macro-level, decline of both gray and white matter in the hippocampus and frontal cortex cause age-related decline in mental flexibility, working memory, attention , and semantic and episodic memory.

Aging is associated with considerable reductions in neuroplasticity (Wong et al., 2021). Long-term potentiation (LTP) is decreased while long-term depression (LTD) is enhanced. Inter-neuronal interaction is also compromised due to impaired glutamatergic transmission (especially in frontal cortical areas), cholinergic hypofunction, and substantial alterations in the dopaminergic system, which together may underlie learning and memory deficits, and impaired cognitive flexibility with advancing age.

Age-related changes in brain structure and function at the level of large-scale neuronal networks are associated with cognitive decline in a variety of domains. For instance, hippocampal synaptic plasticity is reduced throughout aging, and this plasticity is sensitive to adverse (stressful) experiences that can accelerate age-related decline in memory and learning. Aging is also characterized by reduced suppression of the default mode network (DMN) and increased recruitment of regions of the prefrontal cortex. The de-activation of the DMN, typically observed during engagement in a specific task, is less pronounced in later life, while at the same time prefrontal cortex (which is particularly susceptible to declines in gray- and white-matter integrity) is more strongly activated in response to task demands, a phenomenon known as compensatory recruitment. This dual pattern of age-related change is associated with a cognitive shift from favoring explorative to exploitative behaviors in later life.

#### Impaired Neurogenesis: Neuronal Senescence and Stem Cell Exhaustion

In the adult hippocampus, new neurons can be generated from neuronal stem cells in the dentate gyrus (adult hippocampal neurogenesis) (Ming and Song, 2011; Kempermann et al., 2018; Kuhn et al., 2018; Toda et al., 2019; Lucassen et al., 2020). The generation of these new neurons has been implicated in learning and memory in general, and in pattern separation in particular (Clelland et al., 2009; Sahay et al., 2011; Toda and Gage, 2018). Neurogenesis persists during aging (Tobin et al., 2019) but is reduced in the aging brain (Babcock et al., 2021) which may contribute to cognitive deficits (Jessberger and Gage, 2008; Lazarov et al., 2010; Kuhn et al., 2018). Neural stem cell exhaustion maybe important in this aspect since excessive proliferation leads to premature aging (Kippin et al., 2005), and limits the capacity to replace senescent cells. Moreover, during aging, stem cells get exhausted while clearance of senescent cells becomes inefficient, resulting in the accumulation of senescent nerve cells that then may aggravate neuronal damage (Mattson and Arumugam, 2018). The newly generated neurons may die *via* apoptosis, which is triggered by various factors among which telomere shortening (see below) and DNA damage (Collado et al., 2007).

#### Neuronal Integrity, Morphology, Activity, Plasticity, and Communication

Neurons constitute only a proportion of all brain cells; the remaining proportion of brain cells consist of glial cells that have diverse supportive functions (the exact proportions remain debated; Von Bartheld et al., 2016). Oligodendrocytes engulf long axons and form a fatty protective layer. This myelin layer facilitates the propagation of action potentials along the axon, significantly enhances and improves the transmission of information within large coordinated groups of neurons, and is important for learning and memory (Steadman et al., 2020).

In humans, brain weight and volume decrease by about 2% per decade; the sulci become more conspicuous while the ventricles increase in size. These changes entail atrophy but also shrinkage of neuronal soma as part of the process of normal aging. White-matter hyperintensities (small point-like lesions) and other white-matter abnormalities become more numerous with age (Garde et al., 2000; Bennett and Madden, 2014; Liu et al., 2017) and indicate a decrease in myelin. The relationship between these hyperintensities and cognitive decline has the character of a threshold: they initially remain benign, but once their total size reaches a threshold level, cognition begins to deteriorate. Myelination is also highly active in young mice but inhibited in aged mice. Importantly, preventing reduced myelination also prevents memory impairments which may hint at preventing memory impairments in aged mice (Wang et al., 2020).

Regional differences in structural and functional decline adhere to a last-in, first-out principle. The phylogenetically and ontogenetically younger association cortex (especially the prefrontal cortex) is affected more by age-related change than the older primary sensory cortex or subcortical areas (Whalley, 2015), with the exception of the medial temporal lobe, and especially the hippocampus, as reviewed above. The longer it takes for myelin to mature and engulf nerve fibers, the more susceptible the structure in question is to the effects of aging. In the frontal lobe and hippocampus, the gray and white matter decline on average by about 14% and 24%, respectively (Sherwood et al., 2011). Cognitive functions that depend on the frontal lobes, including mental flexibility and inhibition, working memory, selective attention, and divided attention, suffer relatively strongly from age-related decline. Certain forms of memory (such as procedural memory) are more spared than others (such as semantic memory and episodic memory).

Yet, in contrast to what has long been thought, normal aging is not accompanied by a profound decline in neuron number with advancing age in rodents (Rapp and Gallagher, 1996) or humans (West, 1994) (for reviews see Burke and Barnes, 2006). Likewise, hippocampal dendritic morphology and branching is either unchanged, decreased, or may even be increased in aged people and rodents (Burke and Barnes, 2006; Sikora et al., 2021). These effects on dendritic branching may be region-specific since dendritic branching of neurons in the prefrontal cortex appears to be decreasing with age in humans and rodents while remaining relatively intact in primary sensory areas (Burke and Barnes, 2006; Sikora et al., 2021). Also, region-specific changes in density and morphology of dendritic spines have been reported with increased age. The number of spines in the hippocampal CA1 area is relatively stable with advancing age, whereas a reduction in spines has been reported in CA3, subiculum, and cortical neurons (Burke and Barnes, 2006; Sikora et al., 2021).

With increasing age, most electrical properties of neurons remain unaltered (Burke and Barnes, 2006). However, as described above, Ca^2+^ currents and the calcium-dependent afterhyperpolarization are increased in aged neurons. In parallel, LTP, an important experimental model for learning and memory (Nabavi et al., 2014), at peri-threshold stimulation paradigms is decreased in aged rodents, while LTD is enhanced (Burke and Barnes, 2006; Kumar, 2011; Sikora et al., 2021). Such changes in plasticity may underlie cognitive deficits related to aging. In addition, various studies have also suggested that the balance between excitatory/inhibitory transmission may be altered throughout aging, which is critical for network function (Luebke and Rosene, 2003; McQuail et al., 2015; Tran et al., 2018), although region-specific and even intra-region-specific effects may occur (Tran et al., 2018).

Substantial changes in transmitter systems occur during physiological aging, although such changes can berelatively moderate in some of these systems. Glutamate is the major excitatory neurotransmitter in the brain. As discussed before, there may be loss of glutamatergic neurons in cortical areas, while more subtle changes in the hippocampal formation have been observed. At the same time, synaptic plasticity in these neurons is altered, which may relate to a reduction in NMDA receptors (Rosenzweig and Barnes, 2003; Gasiorowska et al., 2021). Like the glutamatergic system, there is also evidence that cholinergic neurons of the basal forebrain exhibit moderate degenerative changes during the course of aging. Substantial loss of cholinergic neurons has been observed in pathologies such as Alzheimer’s disease (Schliebs and Arendt, 2011; Gasiorowska et al., 2021). Cholinergic projections arise from the medial septum, the horizontal and vertical diagonal band of Broca, and the nucleus basalis of Meynert which provide the major cholinergic input to the cerebral cortex and hippocampus. Cholinergic hypofunction during the course of aging may relate to learning and memory impairments with advancing age. The nigrostriatal, mesolimbic, mesocortical, and tuberoinfundibular pathways are the main dopaminergic pathways in the brain. Evidence suggests substantial alterations in the dopaminergic system during normal physiological aging, which involve a reduction in receptors and increased dopamine synthesis (Braskie et al., 2008; Gasiorowska et al., 2021) which relate to altered cognitive flexibility (Berry et al., 2016).

In summary, these studies suggest that throughout normal aging, neuronal integrity is relatively unaffected, whereas spine density, Calcium dysregulation, myelination, and synaptic plasticity are altered, with corresponding age-related cognitive decline.

#### Aberrant Neuronal Network Activity and Connectivity

At more macro levels, the fidelity of neuronal network activity within and between brain regions tends to get compromised during brain aging. The human brain shows considerable reductions in both gray and white matter (Bennett et al., 2010; Maniega et al., 2015; see also Section “Neuronal Integrity, Morphology, Activity, Plasticity, and Communication”), and an associated deepening and widening of sulci and enlargement of the cerebral ventricles (Drayer, 1988). Volumetric analyses have shown age-related decline in gray-matter volume/density in cortical and subcortical areas (Manard et al., 2016; Zsoldos et al., 2018), while diffusion MRI methods have shown age-related changes in white matter connectivity between within and between cortical regions, which are related to age changes in cognition (de Lange et al., 2016; Koini et al., 2018). These age-related changes in brain structure and function are associated with cognitive decline in a variety of domains (Cabeza et al., 2018). In addition, this age-related decline is subject to considerable inter-individual variability. This degradation may proceed relatively subtly and/or gradually in some individuals and more pathologically and/or abruptly (in sudden transitions, such as when a threshold has been transgressed) in others. Interestingly, those individuals who display stable cognitive performance during aging tend to also show less brain decline or pathology (Cabeza et al., 2018).

##### Larger-Scale Neural Systems: the Hippocampal System

As described above, hippocampal neuronal integrity appears to be maintained largely intact throughout aging. However, hippocampal synaptic plasticity is reduced throughout aging. The hippocampus is of particular interest in aging, as it plays a key role in learning and memory consolidation as well as in affective behaviors, mood regulation, and stress regulation.

Interestingly, hippocampal synaptic plasticity and corresponding cognitive decline are sensitive to and affected by exposure to stressors and life events later in life, but also early in life. Stressful experiences enhance the activity of the hypothalamus-pituitary-adrenal axis which results in elevated glucocorticoid levels—cortisol in humans and corticosterone in rodents (de Kloet et al., 2005; Joëls and Baram, 2009). Rodent studies show that chronic social stress and elevated cortisol levels induce cognitive impairment in aged mice and humans (Sterlemann et al., 2010; Bartsch and Wulff, 2015; Lupien et al., 2018). Also, high levels of glucocorticoids may accelerate the aging process, by causing further damage to the hippocampus and increasing the exposure to exaggerated inflammatory responses such as priming of microglia (Landfield et al., 2007; Barrientos et al., 2015a, b). Moreover, stressful experiences in early life also enhance and accelerate the aging process in the hippocampus and the cognitive functions it supports (Lupien et al., 1999; Kumar, 2011; Sousa et al., 2014; Short et al., 2020), while manipulations early in life that reduce corticosterone levels in rodents reduce memory impairments later in life (Meaney et al., 1988; for review see Lesuis et al., 2018). Together, these studies suggest that adverse (stressful) environmental experiences can accelerate age-related decline in hippocampal cognitive function.

While these age changes in hippocampal integrity stand on their own, the hippocampus is also part of the DMN, which will be addressed in the next subsection.

##### Larger-Scale Neural Systems: Prefrontal Cortex and the Default Mode Network

Among the more pronounced age-related changes in functional brain activity at the larger-scale network level are reduced suppression of the DMN and increased recruitment of regions of the prefrontal cortex (Grady, 2012; Andrews-Hanna et al., 2014; Damoiseaux, 2017). In humans, the DMN is an assembly of functionally connected brain regions implicated in the type of self-oriented reflections and ruminations one engages in when not performing a specific mental task. Activation within the DMN generally tends to be suppressed when engaging in a specific task (Buckner et al., 2008), but this suppression becomes less pronounced with aging (Hafkemeijer et al., 2012). This pattern of reduced DMN suppression is accompanied by increased prefrontal recruitment across a wide array of mental tasks (Davis et al., 2008) serving to support cognitive-control processes during task performance (Duncan, 2010; Stokes et al., 2013) in later life (Payer et al., 2006). The prefrontal cortex is particularly susceptible to structural age-related decline in gray matter (Raz et al., 2005) and white matter (Piguet et al., 2009; Yarchoan et al., 2012). At the same time, aging is associated with increased prefrontal activation in response to changing task demands (Cabeza, 2002; Grady, 2012), compensatory recruitment also described as “scaffolding” (Reuter-Lorenz and Cappell, 2008; Park and Reuter-Lorenz, 2009). During aging, prefrontal recruitment and reduced default-network suppression co-occur; with declining cognitive control, the DMN becomes increasingly coupled with lateral prefrontal brain regions (Turner and Spreng, 2015). The age-related reduction of dopaminergic signaling to the hippocampus (Duzel et al., 2010) may alter the functional integrity of the DMN (of which the hippocampus is part; Andrews-Hanna et al., 2010). As a result, the DMN is coupled more tightly with the prefrontal cortex (Spreng and Turner, 2013, 2019). As will be discussed in more detail in Section “Horizons in Human Aging Neuroscience: From Normal Neural Aging to Mental (Fr)Agility”, this pattern of age-related change is associated with a cognitive shift from favoring explorative to exploitative behaviors. Like the hippocampus, the prefrontal cortex is sensitive to stress-exposure later as well as early in life, which both alter its structure and function. In addition, throughout aging, the prefrontal cortex becomes more sensitive to stressors (McEwen and Morrison, 2013).

## How Interventions Can Potentially Accelerate Or Decelerate Brain Aging

**Figure 1:**
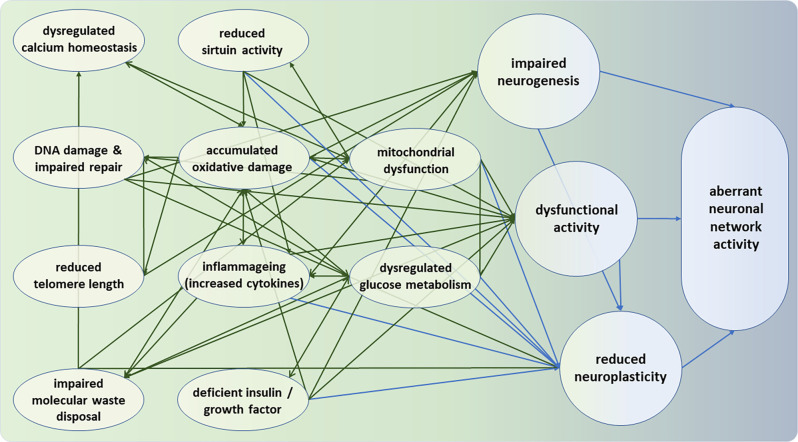
Intra-neural and inter-neural hallmarks of neuronal deterioration. Moving from left (green) to right (blue), the hallmarks represent deficiencies at the molecular, cellular, and neuronal network levels. All intra-neural hallmarks are densely interconnected. It can be seen that the bridge between intra- and inter-neuronal hallmarks of aging appears to be formed by the three nodes of impaired neurogenesis, dysfunctional neural activity, and reduced neuroplasticity. Note that the hallmarks and their interconnections are intended to be representative but not necessarily exhaustive.

It has been suggested that each of the hallmarks of aging reviewed above should fulfill two criteria in order to be causally related to the aging process: its experimental aggravation should accelerate aging; and its experimental amelioration should retard the normal aging process and hence increase healthy lifespan (López-Otín et al., 2013). However, since these hallmarks are largely inter-dependent and often operate in a complex interplay, as discussed, the evidence for these individual criteria is often not consistent. The extensive interconnections between the hallmarks of aging (see Figure 1) raises the suggestion that experimental amelioration of one particular hallmark may impinge not only on others but in fact on the entire complex. Below, we review a limited number of evidence-based interventions that cut across several of these hallmarks and, perhaps as a consequence, appear to exert systematic effects that are relevant in the context of normal neural aging and mental ability and (fr)agility in later life. These interventions can influence age-related change *via* a series of overarching regulators, including more “metabolic” (calorie restriction; exercise) and more “non-metabolic factors” (pharmaca; microbiota; sleep hygiene; prevention of chronic stress). As discussed, age-related cognitive decline is thought to be associated with neural alterations in the aging hippocampus and prefrontal cortex (including increased oxidative stress and neuroinflammation, altered intracellular signaling, and reduced neurogenesis and synaptic plasticity). Since interventions such as calorie restriction, physical exercise, and environmental enrichment have been shown to counteract many of the age-induced alterations in neuronal signaling, structure, and function (Ekstrand et al., 2008; Aksu et al., 2009; Willette et al., 2012; Bettio et al., 2017; Torabi et al., 2019), we will focus on interventions that affect the integrity and function of the hippocampal complex and prefrontal cortex.

### Metabolic Factors

If aging is caused by specific molecular changes, then it should in principle be possible to slow the aging process through targeted interventions. This holds potential for dietary restriction and physical exercise, which retard age-related decline and the incidence of disease, including cancer, neurodegeneration, and cognitive decline at least in rodent studies (Fontana et al., 2010). Energy metabolism is one of the generic dimensions that, when influenced by interventions or naturally occurring conditions, engage an interplay of many of the hallmarks of normal neural aging, and through that interplay can impact brain aging. Calorie restriction on the one hand and physical exercise on the other are among the main interventions known to retard brain aging, *via* their effects on, among others, cellular stress resistance, repair, and growth. Over the course of hundreds of thousands of years, evolutionarily pressures to compete for limited resources resulted in benefited individuals whose brains optimally supported their bodily needs in times of food deprivation (Mattson, 2015). Our present-day patterns of consumption and physical exercise do not align well with those of our ancestors who ate less regularly, consumed calories and fat when they could, and fasting when they could not, and needed to move about more strenuously and persistently in order to obtain food and shelter.

#### Nutrients and Calorie Restriction

Aging is strongly associated with the accumulation of cellular damage. If it were possible to remove known sources of damage from our daily consumption/diet, then it should be possible to reduce the consequences of the damaging process. One obvious starting point is nutrition. Food that promotes maintenance and repair helps to protect the body against accumulated damage; harmful foods add to the burden of damage. Saturated fats and excess sugar may be harmful (Stranahan et al., 2008a, b); fruits and vegetables that are rich in anti-oxidants and fish and olive oils that lower “bad cholesterol” may be protective (Singh and Mishra, 2013). The biochemistry of such damage and protection, and how these nutrients play their part in (preventing) age-related degeneration and disease, are presently being unraveled.

The evidence for the efficacy of commercially available nutritional supplements is inconsistent at best. Stronger evidence derives from randomized controlled trials in humans and, especially experimental studies in animals, on calorie restriction. These animal experiments allow for studying the effects of nutrition manipulations on the hallmarks of normal neural aging. Many of these hallmarks are mitigated, for instance, when animals are fed/fasted intermittently, compared to animals fed *ad libitum* (López-Otin et al., 2016; Mattison and Vaughan, 2017). Calorie restriction stimulates neuronal mitochondrial biogenesis either directly (Martin-Montalvo and de Cabo, 2013; Hood et al., 2015) or through activating neural signaling pathways (Cheng et al., 2012; Raefsky and Mattson, 2017; Mattson et al., 2018), and mitigates the neuronal mitochondrial aging effects of oxidative damage and Ca^2+^ homeostasis (Mattson and Arumugam, 2018). Rats or mice maintained on low-fat and/or low-sugar diets exhibit reductions in oxidative damage (Elahi et al., 2016), autophagy (Li et al., 2017), and neuroinflammation (Jayaraman et al., 2014), and neuronal Ca^2+^ homeostasis (Thibault et al., 2013). Excessive energy intake compromises the ability of neurons to respond adaptively to oxidative stress, as indicated by reduced expression of SIRT1 (Heyward et al., 2016) and BDNF (Stranahan et al., 2008b). The sirtuin SIRT3 is involved in mediating the beneficial effects of dietary restriction, due to the deacetylation of mitochondrial proteins (Someya et al., 2010).

The cellular complex called the mechanistic target of rapamycin (mTOR) is a protein kinase that in mammals functions as the key regulator of cellular growth and metabolism in response to nutrient and hormonal cues (Stanfel et al., 2009). When mTOR is in “production mode”, the neuron produces proteins that serve as building blocks for growth. When mTOR is in “maintenance mode”, damaged proteins are repaired and reused, and accumulated waste is removed (Höhn et al., 2013). Our body responds to sugar consumption by producing the glucose transporter insulin. Insulin activates the growth factor IGF-I, which signals mTOR to convert glucose into protein, and hence growth. Conversely, calorie restriction results in the blocking of mTOR, so that protein production and hence the growth of cells (including tumor cells) is arrested. The repair of damaged proteins and the removal of waste invigorates the nerve cells, making them more resistant to stress and less susceptible to damage during aging (Harrison et al., 2009; Anisimov et al., 2011). Interestingly, the mTOR inhibitor rapamycin delays multiple aspects of aging in mice (Wilkinson et al., 2012; Johnson et al., 2013).

Excessive energy intake has been reported to accelerate hippocampal atrophy (Cherbuin et al., 2015), whereas rodents maintained on low-fat and/or low-sugar diets exhibit improved regulation of neuronal network activity (Margineanu et al., 1998). Rats on a high-fat and high-sugar diet exhibit elevated markers of oxidative stress and inflammation in their hippocampus, and perform worse on a place recognition task (Beilharz et al., 2014). Calorie restriction and intermittent fasting delay structural and functional age-related decline in rodents and monkeys (Duan et al., 2003; Willette et al., 2010; Mattison et al., 2012). Importantly, in humans, body mass index (BMI) is inversely associated with glucose utilization in the prefrontal cortex (Volkow et al., 2009) and neuroimaging studies document reduced gray-matter volumes and white-matter integrity in multiple brain regions, and reduced functional connectivity between brain regions in obese individuals (Debette et al., 2014; Kullmann et al., 2015).

#### Exercise

Calorie restriction and physical exercise are—at least in part—two faces of the same coin. While the metabolic challenges they pose to the brain differ, they both activate pathways that prepare the cells to recuperate during recovery periods, such as rest and sleep (Mattson et al., 2018), and they both incite adaptive cellular responses that enhance neuroplasticity and tolerance of metabolic stress in similar fashion (Mattson and Arumugam, 2018). Overindulgent and sedentary individuals may likewise be more prone to impaired brain function and accelerated neurodegenerative aging.

Sedentary lifestyles are in a way unnatural. When people stop moving and do not challenge their brains to organize action to solve the problems of navigating a fickle environment, this may amplify the accrual of cellular damage. Exercise takes on a greater significance as we grow old, in retarding (or even reverting) some of the accumulating molecular deterioration. Neural signaling pathways activated by exercise can also stimulate the generation, stress resistance, and quality control of the mitochondria in nerve cells (Cheng et al., 2012; Raefsky and Mattson, 2017). Driving our cells to their energetic limits may force selection of mitochondria whose DNA is not damaged by free radicals (Kirkwood, 2001). Beneficial effects further include the stabilization of neuronal calcium homeostasis, the suppression of neuroinflammation and oxidative stress, and the regulation of DNA repair, autophagy, and neurotrophic factor signaling (Mattson et al., 2018). Exercise has also been reported to enhance telomere length and the efficacy of astrocytes in providing the energy for synaptic plasticity (Morita et al., 2019).

In our ancestral past, physical activity was tightly associated with pathfinding and spatial memory. Hence, exercise stimulates hippocampal neurochemical activity, increases the hippocampal volume (Erickson et al., 2011), neurogenesis (van Praag et al., 1999a, b), and improves memory function in older animals, including humans (Intlekofer and Cotman, 2013; Boraxbekk et al., 2016; Chirles et al., 2017; Voss et al., 2019). A meta-analysis of neuroimaging studies in humans (Ji et al., 2021) showed that physical exercise results in structural and functional changes in the hippocampus and prefrontal cortex, changes that were consistently associated with less pronounced cognitive decline.

### Nonmetabolic Factors

Factors that do not target primarily the metabolic system can affect some of the hallmarks of normal neural aging through other means. Here we will briefly discuss the effects of microglia replacement, microbiota, certain chemicals, sleep hygiene, chronic stress, and cognitive exercise.

#### Sleep

During sleep, cell repair and immune responses are boosted (Bollinger et al., 2010). Sleep loss (either in terms of hours of sleep, sleep quality, or intermittent periods of non-sleep) can counter these processes, and is known to increase the allostatic burden and hence add to the cumulative effects of chronic stress (Palagini et al., 2013). Moreover, during sleep, amyloid sediments are removed *via* the cerebrospinal fluid through channels that expand while sleeping; sleep loss prevents this (Xie et al., 2019).

Among the brain areas most affected by sleep deprivation, the hippocampus takes a prominent place (Havekes and Abel, 2017). As a neuromodulator for the hippocampus, acetylcholine plays a role in memory consolidation; during non-REM sleep, acetylcholine levels drop which promotes the consolidation of memories, which benefits in particular procedural and motor learning (Inayat et al., 2020). In older adults, decreased production of melatonin and weaker circadian rhythms impact the ability to fall asleep and subsequently the quality, duration, and fragmentation of sleep; intermittent periods of being awake are experienced as particularly frustrating. Disturbed sleep among older adults promotes neurodegeneration, especially in the hippocampus, and can amplify memory problems and depression (Yaffe et al., 2014). The production of melatonin, a hormone excreted by the pineal gland that promotes falling asleep after dark, declines substantially among older adults; melatonin supplements can to some extent alleviate age-related sleep problems (Poeggeler, 2005).

#### Chronic Stress

Chronic stress, such as associated with burnout, mental trauma, chronic pain, prolonged intensive care for a parent or spouse, or low social cohesion, is associated with reduced hippocampal function and cognitive decline, but also with reduced telomere length (Lupien et al., 1998; Epel and Prather, 2018). Moreover, early life stress may also influence telomere length (Price et al., 2013). Mindfulness meditation, which reduces stress, may enhance the activities of telomerase and can result in lengthened telomeres (Schutte et al., 2020). The mechanisms by which chronic uncontrolled stress compromise the neuronal stress response are similar to that of metabolic morbidity, and may involve an acceleration of aging processes (Sapolsky, 1999; Hekimi et al., 2011).

Aging is also associated with increased loneliness. In Amsterdam, with a total population of 800,000 inhabitants, 30,000 seniors reported in 2020 that they felt lonely and did not have a meaningful conversation more than once a week. Although the effects of loneliness on chronic stress may be subject to individual differences, loneliness can lead to an increase in proinflammatory cytokines (Smith et al., 2020), and* vice versa*, inflammation can modulate social processes (Moieni and Eisenberger, 2018), reduce the production of antiviral interferons (Capitanio et al., 2019), hyperactivation of the HPA-axis (Campagne, 2019), and can neutralize the beneficial effects of exercise on neurogenesis (Kozareva et al., 2018). Lonely people have reduced concentrations of dopamine receptors in the dorsal and ventral striatum, reducing the pleasures and rewards of social interaction (Inagaki et al., 2016; Matthews et al., 2016). Listening to music, especially together with others, stimulates the release of oxytocin (Chanda and Levitin, 2013). In some older individuals, medicinal selective serotonin re-uptake inhibitors (SSRI’s) may reduce social anxiety and promote social contact, although these effects may depend on expectancy effects (Hjorth et al., 2021). Volunteer activities promote social connectedness, as well as cognitive flexibility, and increased activation of prefrontal cortex.

#### Microbiome

Accumulating evidence suggests that gut microbiome composition changes with age. The gut microbiome shapes among others the function of the host immune system, in particular *via* microglia in the brain (Mohajeri et al., 2018; Erny and Prinz, 2020) and exerts systemic metabolic effects (Ottaviani et al., 2011). Certain gut bacteria as well as the fecal dopamine metabolite 3,4-dihydroxyphenylacetic acid are positively associated with quality of life, while other gut bacteria and fecal microbial γ-aminobutyric acid metabolites are negatively associated with depression (Valles-Colomer et al., 2019). Age-related changes in the microbiota and immune system are associated with the accumulation of nerve cellular waste and cognitive decline (Scott et al., 2017), and specific metabolites act as signaling molecules to the brain to actively regulate longevity (Zhou et al., 2021).

A relationship has been established between diet, microbiota, and health status, pointing to a role for diet-driven microbiota alterations in varying rates of age-related health decline (Claesson et al., 2012; Kim et al., 2018). The effects of aging on the brain were ameliorated in aging mice through a microbiota-targeted diet (Boehme et al., 2020), and transplanting the microbiome from the feces of young mice into the intestines of old mice resulted in the reversal of age changes in the immune system, inflammation, and hippocampal metabolic waste while improving their memory and learning skills (Boehme et al., 2021).

#### Microglia

Microglia, the innate immune effectors of the brain, are involved in the regulation of inflammation, synaptic connectivity, programmed cell death, and autophagy of cell debris; microglial senescence is associated with a chronic inflammation phenotype and with the loss of neuroprotective functions that lead to a greater susceptibility to the neurodegenerative diseases of aging (Gabuzda and Yankner, 2013; Mecca et al., 2018). Replacing microglia in aged mice brains with new microglia, using microglial repopulation *via* pharmacological inhibition of the colony-stimulating factor 1 receptor (CSF1R), rejuvenated physical microglial cell densities and morphologies, and improved spatial memory (Elmore et al., 2018). In the latter study, age-related changes in hippocampal neuronal complexity were reversed, while neurogenesis, dendritic spine densities, and LTP were increased. It remains to be established whether such approaches might work in humans as well.

#### Pharmacology

In prematurely telomerase-deficient aged rodents, aging can be retarded or even reverted by telomerase activation (Jaskelioff et al., 2011; Maynard et al., 2015). Moreover, systemic viral transduction of telomerase retards normal physiological aging in adult wild-type mice, presumably through countering the telomere shortening that typically occurs with age (Bernardes de Jesus et al., 2012). In humans, supplements, pills, or injections containing telomerase are not adviced because of the risk of promoting cancer cell growth (Epel et al., 2004).

Metformin, a medicine against diabetes, enhances the activity of the enzyme adenosine monophosphate-activated protein kinase (AMPK), which can mimic the effects of calorie restriction, can reduce the activity of IGF-1, and can reduce the toxic waste of senescent cells. In mice and humans, metformin reduces inflammation and oxidative stress as well as cognitive decline (Garg et al., 2017).

A recent surge of interest has focused on dietary ergothioneine, a compound that accumulates at high levels in the body from diet (in particular in mushrooms) and that was recently approved by the U.S. Food and Drug Administration as a supplement. In humans, plasma levels of ergothioneine decline with age (Cheah and Halliwell, 2021). In a *C. elegans* model of abnormal accumulation of β-amyloid peptide (Aβ), ergothioneine incurs dose-dependent reductions in Aβ-oligomerization (thus affecting oxidative stress, inflammation, and mitochondrial dysfunction) and extends longevity (Cheah et al., 2019). In mouse models, ergothioneine counters the negative effects of hydrogen peroxide (H_2_O_2_)-induced neurotoxicity on ROS and mitochondrial membrane potential in hippocampal neurons, suggesting ergothioneine’s potential as an antioxidant neuroprotectant in hippocampal neurons (Kushairi et al., 2020). In a study on humans with and without dementia, lower ergothioneine levels were also associated with white-matter hyperintensities and reduced global cortical thickness and hippocampal volumes (Wu et al., 2021).

#### Cognitive Training

As reviewed above, exercise counters the neural effects of aging at intra- and inter-neuronal levels, with corresponding improvements in cognitive function. Possibly, such effects may be triggered also by cognitive exercise in ways reminiscent of those of physical exercise. Indeed, in old mice placed in an environment enriched with running wheels, tunnels, and a variety of other challenges, neurogenesis increased by 15%, an increase that was particularly pronounced in the hippocampus (Kempermann et al., 2002).

Experience-dependent plasticity has also been observed in humans. Novices who were trained for three months in juggling with three balls showed increased gray matter density in brain regions involved in visual motion perception and eye–hand coordination (Draganski et al., 2004; Gerber et al., 2014). Older adults showed similar changes, along with increased hippocampal gray matter (Boyke et al., 2008). A recent study showed increases in white-matter fractional anisotropy in the somatosensory and visual cortex that covaried with behavioral improvement among novices during 8 months of training on tactile braille reading (Molendowska et al., 2021). London taxi drivers, whose jobs require them to memorize numerous complicated routes and locations, were found to have hippocampi whose size was directly proportional to their number of years of experience and, hence their age (Maguire et al., 2000).

Beyond these specific types of expertise, training of more generic cognitive skills has not yielded consistent effects. For instance, a recent meta-analysis of 43 studies on commercial training programs among healthy older adults (including a large-scale study from our own lab that we consider state-of-the art; Buitenweg et al., 2019) concluded that there is currently insufficient empirical evidence to support that such training can improve memory, general cognition, or everyday functioning (Nguyen et al., 2021). As to the efficacy of working-memory training, a hot debate is still raging (Karbach and Verhaeghen, 2014; Sala et al., 2019; Hou et al., 2020). In general, although cognitive training (especially of specific skills) might stimulate the genesis of neurons, myelin, and synapses, future research will need to establish whether cognitive training in older adults incurs direct effects on (multiple) other hallmarks of normal neural aging.

### Summary

We reviewed several interventions that potentially influence age-related change *via* metabolic (calorie restriction; exercise) and nonmetabolic factors (chemicals; microbiota; sleep hygiene; prevention of chronic stress). Energy metabolism is a generic dimension that, when influenced by interventions or naturally occurring conditions, engages an interplay of many of the hallmarks of normal neural aging, and through that interplay can impact brain aging. Calorie restriction and physical exercise can retard brain aging *via* their effects on cellular stress resistance, repair, and growth. Normal aging is characterized by compromised neuronal glucose metabolism; interventions that bolster this metabolism can potentially mitigate several hallmarks of brain aging and their interactions, and may thereby delay or slow the processes of normal neural aging.

Many of the intra-neuronal hallmarks of aging are affected by calorie restriction, which stimulates neuronal mitochondrial biogenesis, blocks the mTOR signaling pathway, and mitigates the neuronal mitochondrial aging effects of oxidative damage and Ca^2+^ handling, autophagy, and neuroinflammation. Excessive energy intake compromises the ability of neurons to respond adaptively to oxidative stress. Intermittent fasting promotes the regulation of cellular metabolism, cellular signaling, DNA repair, and mitochondrial function in the hippocampus and prefrontal cortex. Physical exercise counters the accrual of cellular damage, retards or reverts some of the accumulating molecular deterioration, stimulates mitochondrial stress resistance and quality control, stabilizes neuronal calcium homeostasis, suppresses neuroinflammation and oxidative stress, improves the regulation of DNA repair, autophagy, and neurotrophic factor signaling, and serves to enhance telomere length and the efficacy of astrocytes in providing the energy for synaptic plasticity.

Nonmetabolic factors that affect multiple hallmarks of normal neural aging include chemicals, microbiota, sleep hygiene, and chronic stress. Cell repair, waste clearance, and immune responses are intensified during sleep, while the allostatic burden is increased by sleep loss. Disturbed sleep among older adults promotes neurodegeneration, especially in the hippocampus, and can amplify memory problems and depression. Chronic stress is associated with reduced telomere length, compromised neuronal stress response, and reduced hippocampal function. Loneliness increases proinflammatory cytokines; inflammation causes a reduction in glutamate and antiviral interferons, and neutralizes the beneficial effects of exercise on neurogenesis. Consistent evidence for the efficacy of cognitive training however has not yet been obtained.

The potential interventions that are discussed above and their targets may provide novel avenues for enhancing healthy aging, improve mental wellbeing and reduce mental fragility as will be discussed in the next section.

## Horizons in Human Aging Neuroscience: from Normal Neural Aging to Mental (Fr)Agility

We reviewed the complex of hallmarks of neurobiological aging vis-à-vis the “normal” deterioration of mental ability and agility. We aimed to reconcile literature on the cellular and molecular mechanisms, processes, and antecedents of neural aging, on the one hand, and on functional and structural integrity (activation, connectivity, and plasticity) within and between large-scale neural networks in its relationships to age-related cognitive decline, on the other hand. We purposefully focused on *normal* aging across levels: from molecular to cellular and network changes to behavioral/mental ability and agility. In particular, normal neural aging in the complex interplay of neural factors that tap into the hippocampal system was shown to connect in intricate ways to declines in memory function.

### Connecting Intra-neural and Inter-neural Hallmarks of Neuronal Deterioration

Section “How Human Brains Age: Intra-neural and Inter-neural Hallmarks of Neuronal Deterioration” highlights various hallmarks of intra-neural and inter-neural aging, and interconnections between these hallmarks, that together form a multifaceted system that determines the aging phenotype. This pattern can be summarized as follows, along the dimensions of metabolism on the one hand, and damage and waste on the other.

Reduced mitochondrial fitness is among the centrally connected factors in the complex responsible for normal neural aging, relating to glucose metabolism, cellular Ca^2+^ homeostasis, protein deacetylases of sirtuins, accumulated oxidative stress, reactive oxygen species, and damaged DNA. Moreover, as described in Section “Molecular and Cellular Hallmarks of Brain Aging”, glucose metabolism, calcium homeostasis, DNA stability and repair, aggregation of proteins, inflammageing, have been involved in the aging process. As such, there is ample evidence that in the course of aging, the brain is subjected to various metabolic and oxidative challenges that render neurons vulnerable to impaired function. At the same time, the potential capacity of appropriate defense mechanisms is reduced. As shown in Figure 1, these factors can interact and in synergy determine neuronal function, and ultimately cognitive function by altering among others neurogenesis, synaptic plasticity, and neuronal network activity (Geerligs et al., 2015).

The figure illustrates that the bridge between intra- and inter-neuronal hallmarks of aging appears is formed largely by dysfunctional neural activity and reduced neuroplasticity, and, to a lesser extent, by impaired neurogenesis. Some implications of this apparent conduit will be discussed vis-à-vis interventions in Section “Interventions” below.

#### The Hallmarks of Neural Aging: A Complex Pattern of Interactions

We discussed that molecular and cellular mechanisms and antecedents of normal neural aging form a complex of intricately interconnected factors that engage in complex interactions, with an apparent bridging role for dysfunctional neural activity and reduced neuroplasticity. Importantly, we suggest that mental aging cannot be reduced to a single neurobiological cause, but should instead be considered in terms of systems that contain reciprocally causally connected symptoms. Beyond studying the effects of these factors in isolation, considerable insight may be gained by studying the larger picture that entails a representative collection of such factors and their interactions, ranging from molecular to network level.

For instance, mitochondrial and bioenergetic changes observed with advancing age are reminiscent of those observed with Alzheimer’s disease, although the latter tend to be of greater magnitude (Swerdlow et al., 2017). The mitochondrial cascade hypothesis (Swerdlow and Khan, 2004) places mitochondrial dysfunction at the apex of the AD pathology pyramid and reconciles seemingly disparate histopathologic and pathophysiologic features. Oxidative mitochondrial DNA, RNA, lipid, and protein damage amplifies ROS production and triggers three events: (1) a reset response in which cells respond to elevated ROS by generating the beta-sheet protein, beta amyloid, which further perturbs mitochondrial function, (2) a removal response in which compromised cells are purged *via* programmed cell death mechanisms, and (3) a replace response in which neuronal progenitors unsuccessfully attempt to re-enter the cell cycle, with resultant aneuploidy, tau phosphorylation, and neurofibrillary tangle formation (Swerdlow and Khan, 2004). Simulations with a mathematical causal model of the cascade hypothesis demonstrated the feasibility of this model (Jack et al., 2010; Petrella et al., 2019). We propose that the mitochondrial cascade hypothesis may potentially be extended to normal aging. Interestingly, in cascade models, the decline in one subsystem may trigger the decline in other, otherwise thus far stable subsystems (Brummitt et al., 2015). Thus, mitochondrial dysfunction, once exceeding a certain threshold, may begin to show a decline, and then pull along other hallmarks of neural aging in its “fall”, thus triggering a cascade that may at some point also incur a decline of cognitive functions supported by neuronal ensembles and networks. It is well possible that multiple hallmarks show thresholds below which deterioration does not yet lead to significant dysfunction, and that transgressing that threshold is triggered only by the “fall” of other hallmarks. It may also well be that a cascade of normal neural aging is triggered not by mitochondrial dysfunction but by some other hallmark instead. It will be worthwhile to investigate if there is any evidence for such a cascade, and if such a cascade at the neural level relates to (a cascade) of cognitive decline and, eventually, a decline in mental well-being.

#### Interventions

Assessing the relationships between compromised neuronal function and network integrity can help in understanding the connection between neurobiological aging at the molecular and cellular levels on the one hand, and the decline of complex cognitive functions on the other. This may help to develop novel avenues for potential interventions that target the intra- and inter-neuronal hallmarks of aging, as described in Section “How Interventions Can Potentially Accelerate Or Decelerate Brainaging”.

We observed that impaired neurogenesis, dysfunctional neural activity, and reduced neuroplasticity are important in bridging intra- and inter-neuronal hallmarks of aging. Yet, this does not necessarily imply that interventions geared toward reducing the negative effects of aging should focus exclusively (or even primarily) on these three bridge-nodes directly. Interventions that ameliorate the effects of normal aging on the intra-neuronal hallmarks may well serve to counter impaired neurogenesis, dysfunctional neural activity, and reduced neuroplasticity in later life.

We reviewed a number of interventions that hold the potential to targeting the complex pattern of interaction among the hallmarks of neural aging at molecular, cellular, network, and cognitive levels. We propose that the way forward is in interventions that target multiple neural hallmarks of aging. For instance, the regular metabolic switching that results from the combination of intermittent fasting and exercise can potentially counteract the complex hallmarks of neural decline and their interplay. Whether working with humans or animal models, the research agenda for the next decades should focus on a broader understanding of additional and novel noninvasive interventions that hold the potential to target not just one single hallmark of aging, but possibly (various hallmarks of) the entire cascade.

While some of the interventions appear to hold potential in postponing or preventing decline, or even restoring functionality, the extent of such malleability and the dynamic range of improvement remains to be determined. Although true biological rejuvenation is once more on the scientific radar (see Section “Outlook” below), most interventions hold more modest promise. But even the most modest improvements may count.

#### Large-Scale Neuronal Networks: Beginning to Bridge Normal Neural and Cognitive Aging

As reviewed in Section “How Human Brains Age: Intra-neural and Inter-neural Hallmarks of Neuronal Deterioration”, the patterns of age-related neural change at the molecular and cellular levels are accompanied by alterations in synaptic plasticity and inter-neuronal communication. Individual differences in structural and functional neuronal network erosion correlate strongly with individual differences in cognitive decline. For example, reduced synaptic plasticity in the hippocampus is associated with declining memory functions, as well as with effects of stress-induced elevations in glucocorticoid levels which in turn alter the hippocampal structure, inflammation, and further cognitive impairments.

Compromised inter-neuronal communication prominently comes to expression in compromised neuronal network activity within and between brain regions. For instance, in older adulthood, the DMN and prefrontal cortex become increasingly coupled. Deactivation of the DMN, as observed when an individual engages in a specific mental activity, becomes less pronounced with aging, a pattern that is accompanied by compensatory prefrontal recruitment. These patterns are associated with marked changes in whether mental tasks are approached from an exploitative or an exploratory cognitive mode (Hills et al., 2015; Baror and Bar, 2016). Exploitative behaviors entail a tendency to rely on prior knowledge and avoid novelty and uncertainty; exploratory behavior tends to favor novel situations over previous knowledge (Schwartenbeck et al., 2013). When the capacity for cognitive control is limited, exploitation of prior knowledge tends to be favored over novelty seeking (Baror and Bar, 2016). Age-related declines in cognitive control are associated with a shift in balance away from exploratory toward exploitative cognitive modes in later life (Spreng and Turner, 2019). Likewise, aging is associated with a shift from goal-directed to routine action (de Wit et al., 2012, 2014) and with a shift from flexibility to stability (Fallon et al., 2013; Berry et al., 2016). Socioemotional selectivity theory (Carstensen et al., 2003) argues that an increasing awareness of the finiteness of time causes aging individuals to favor exploitation of affectively salient goals that provide immediate personal satisfaction over exploration of new knowledge and behaviors with uncertain outcomes.

Notably, the shift toward a more exploitative cognitive mode in later life is associated with increased prefrontal activation and reduced DMN deactivation as well as reductions in hippocampal structure and function (Buckner et al., 2004). The age-related reduction of striatal dopaminergic signaling to the hippocampus (Duzel et al., 2010) has been argued to alter the functional integrity of the hippocampus and of the DMN (of which it is part) more broadly (Andrews-Hanna et al., 2010). As a result, the DMN is coupled more tightly with the prefrontal cortex, promoting exploitation rather than exploration (Spreng and Turner, 2019). Indeed, reduced dopamine signaling within striatal-hippocampal circuitry is associated with age-related reductions in exploratory or novelty-seeking behaviors (Duzel et al., 2010).

#### Individual Differences

The borderland between healthy and pathological neural aging is a twilight zone since it is not always possible to discern clear demarcations between the two. Individuals who display hypertension, diabetes, obesity, or (other) cardiovascular risk factors may well be at risk for developing pathologies such as neurodegenerative disorders or depression, but the determinants of who will and who will not develop pathology remain somewhat elusive. In this review, we have focused on healthy neural aging in the sense of individuals not diagnosed with neurological pathology but we must acknowledge that the distinction is often obscure.

Furthermore, some of the hallmarks of neural aging reviewed here are also hallmarks of pathologies that are not directly related to the aging process. For example, mitochondrial dysfunction may be associated with metabolic complications such as hypertension or obesity, regardless of whether such dysfunction is primarily age-related or not, making it difficult to discern whether such complications are specific to aging or pertain to other alterations. Thus athe focus here was on hallmarks of neural alteration that (jointly) contribute to an age-related neural decline in relation to age-related cognitive decline.

Moreover, the complex interactions between the hallmarks of neural aging that determine cognitive and functional alterations may develop differentially between individuals, depending on for instance experienced environmental challenges (such as stress-exposure) and defense mechanisms which may be more genomic in nature. For example, adverse experiences early in life have been related to cognitive alterations during the course of aging. However, adverse early life experiences such as stress or famine have also been associated with an increased risk of developing cardiovascular disorders later in life (Meaney et al., 2007), which also can have a genetic component (Kathiresan and Srivastava, 2012). Although it is beyond the scope of this review, such alterations may contribute to altered brain and cognitive function and may even be a risk factor for dementia (de Bruijn and Ikram, 2014).

#### Outlook

In summary, we want to emphasize that ultimately, the field should aim for understanding the neurocognitive mechanisms underlying healthy aging, and designing much-needed interventions that promote, quality of life among older adults. One encouraging example, although correlational in nature, and as of yet not focusing on aging, may cast light on the next steps ahead. Surveying a large microbiome population cohort (Flemish Gut Flora Project, *n* = 1,054), Valles-Colomer et al. (2019) analyzed fecal metagenomes and observed that the synthesis potential of certain microbiota metabolites as well as certain bacteria are positively associated with mental quality of life (as reviewed in more detail in Section “Microbiome”). Such studies may help advance our understanding of the relationship between physiological factors (in this case gut microbial metabolism and its metabolite effects on the brain) and mental well-being among older adults.

## Author Contributions

KRR and HJK together wrote this article. All authors contributed to the article and approved the submitted version.

## Conflict of Interest

The authors declare that the research was conducted in the absence of any commercial or financial relationships that could be construed as a potential conflict of interest.

## Publisher’s Note

All claims expressed in this article are solely those of the authors and do not necessarily represent those of their affiliated organizations, or those of the publisher, the editors and the reviewers. Any product that may be evaluated in this article, or claim that may be made by its manufacturer, is not guaranteed or endorsed by the publisher.

## Footnotes

^1^ https://www.who.int/philippines/news/q-a-detail/healthy-ageing-and-functional-ability#:~:text=WHO%20defines%20healthy%20ageing%20as,they%20have%20reason%20to%20value
